# The Effect of Inversion Time on the Relationship Between Iron Oxide Nanoparticles Concentration and Signal Intensity in T1-Weighted MR Images

**DOI:** 10.5812/iranjradiol.12667

**Published:** 2014-05-15

**Authors:** Hodaiseh Saharkhiz, Nahideh Gharehaghaji, Mahmood Nazarpoor, Asghar Mesbahi, Masoud Pourissa

**Affiliations:** 1 Department of Medical Physics, Faculty of Medicine, Tabriz University of Medical Sciences, Tabriz, Iran; 2 Student Research Committee, School of Medicine, Tabriz University of Medical Sciences, Tabriz, Iran; 3 Department of Radiology, School of Paramedicine, Tabriz University of Medical Sciences, Tabriz, Iran; 4 Department of Radiology, School of Medicine, Tabriz University of Medical Sciences, Tabriz, Iran

**Keywords:** Magnetic Resonance Imaging, Inversion Recovery, Ferrosoferric Oxide, Nanoparticles

## Abstract

**Background:**

Magnetic nanoparticles have been widely applied in recent years for biomedical applications. Signal intensity (SI) of magnetic resonance (MR) images depends on the concentration of nanoparticles. It is important to find the minimum concentration of iron oxide nanoparticles that produces maximum SI and determines the minimum injection dose for clinical studies.

**Objectives:**

This study was performed to determine the relationship between the iron oxide nanoparticle concentration and SI using inversion recovery (IR) sequence in T_1_-weighted MR images.

**Materials and Methods:**

Different concentrations of carboxydextran-coated iron oxide nanoparticles 20 nm in size were prepared. In vitro MR imaging was performed with inversion times (TI) of 100-400 ms (interval of 20 ms) and IR Turbo-FLASH (Turbo fast low angle shot) pulse sequence using a 1.5 T MRI system. Then the SI produced by each concentration of nanoparticles was measured and the minimum nanoparticle concentration that led to the maximum SI was determined. Coil non-uniformity was also considered for measuring the accurate SI of each image.

**Results:**

The results indicate that SI depended on the concentration of nanoparticles and TI. In addition, SI increased by increasing the TIs ranging from 200 to 400 ms for all studied concentrations. The linear relationship between the nanoparticle concentrations and SI that gave a square correlation coefficient (R^2^) equal to 0.99 was seen up to 76.83 µmol Fe/L in 400 ms for long TI and 239.16 µmol Fe/L in 200 ms for short TI.

**Conclusions:**

TI is an important parameter to consider in the relationship between SI and nanoparticle concentrations. An increase in TI leads to a decrease in the range of linearity.

## 1. Background

Magnetic nanoparticles have been widely applied in recent years for biomedical applications such as magnetic resonance imaging (MRI) (1, 2). Among various types of magnetic nanoparticles, iron oxide nanoparticles have received increased attention as an MRI contrast agent due to their biocompatibility, superparamagnetic properties and high magnetic moment that generates microscopic field inhomogeneities (3, 4). These nanoparticles have been used for diagnosing various diseases such as cancers in primary stages (5), neurodegenerative diseases (e.g. multiple sclerosis, stroke) (6, 7), inflammatory diseases (e.g. rheumatoid arthritis, atherosclerosis) (8) and cardiovascular diseases as well as tissue perfusion studies (5, 9, 10). The nanoparticles have been used as an MRI negative contrast agent in high concentrations for cancer detection (11, 12). On the other hand, the T_1_ effect of these particles appears in low concentrations and this feature has been used for the study of the heart and blood vessels and tissue perfusion studies (5, 13). The T_1_ effect is enhanced using T_1_-weighted images and leads to achieve high signal intensity (SI) while negative contrast of the particles is enhanced in T_2_-weighted images and produces low SI and artifact due to blood, air and the partial volume effect. Among various MRI sequences for obtaining the T_1_ effect, inversion recovery (IR) is an appropriate sequence to show the small amounts of the contrast agent and to suppress the background tissue and blood signals (9). Inversion time (TI) selection in IR sequence has an important role in achieving adequate diagnostic information. It has been shown that the dose of the contrast agent is one of the main effective parameters on TI length (14).

The SI of MR images depends strongly on the concentration of the nanoparticles. Previous researchers have studied the relationship between iron oxide nanoparticle concentration and SI for in vitro and in vivo situations. The efficacy of ultrasmall superparamagnetic iron oxide (USPIO) nanoparticles as a positive contrast agent for perfusion imaging was evaluated by Chambon et al. (15). They also obtained maximum signal enhancement in vitro with USPIO nanoparticles using spin echo (TR: 500 ms/TE: 22 ms) pulse sequence at the concentration of 200 µmol Fe/L. Additionally, Canet et al. evaluated the relationship between SI and different concentrations of superparamagnetic iron oxide (SPIO) nanoparticles in vitro using Turbo-FLASH (Turbo fast low angle shot) sequence and found the linear portion of the curve up to 200 µmol Fe/L (16). Moreover, Reimer et al. evaluated myocardial perfusion and MR angiography of the chest using different concentrations of SH U 555 C nanoparticles and T_1_-weighted Turbo-FLASH (with TI of 200 ms) and 3D FLASH sequences (17). In this study, they reported the capability of these nanoparticles for depiction of blood vessels in the chest at first pass MR angiography and for cardiac perfusion at the highest applied dose (40 µmol Fe/kg). The effect of TI on the linear relationship between different concentrations of Gd-DTPA and SI was evaluated in vitro(18). Moreover, some researchers have used IR sequences with different concentrations of SPIO or USPIO nanoparticles in vitro for the determination of T_1_ relaxation time (19, 20). However, according to our knowledge, there is no in vitro study showing the effect of TI on the linear relationship between different concentrations of iron oxide nanoparticles and SI. On the other hand, determination of the minimum concentration of iron oxide nanoparticles that produces maximum SI can provide the minimum injection dose for clinical studies. Ultimately, it helps to achieve appropriate image quality and accurate diagnosis.

## 2. Objectives

The purpose of this study was to evaluate the effect of TI on the linear relationship between iron oxide nanoparticle concentrations and SI in T_1_-weighted images using IR pulse sequence.

## 3. Materials and Methods

### 3.1. Contrast Agent

The used contrast agent was Nanomag-D-spio nanoparticles (Micromod Partikeltechnologie GmbH, Rostock, Germany) with a mean diameter of 20 nm. These iron oxide nanoparticles were carboxydextran coated. The concentration of Fe in ferro-fluid was 1.29 mg/mL that was measured using atomic absorption spectrometer (analytik jena, novAA® 400P, Germany). Then 1.8 mL of the ferro-fluid was diluted using 48.2 mL deionized water and 50 mL of the highest concentration (500 µmol Fe/L) was prepared. Other concentrations were prepared using dilution of the concentration of 500 µmol Fe/L with appropriate volumes of deionized water. Prepared concentrations of the nanoparticles included 0 to 100 µmol Fe/L with 5 µmol Fe/L increments and 100 to 500 µmol Fe/L with 100 µmol Fe/L increments.

Although the diluted nanoparticle samples had low concentrations and the probability of precipitation of the nanoparticles was low, the vials were put in ultrasonic bath (Alex Machine, Turkey) for 20 minutes before MR imaging to uniform the disperse of the nanoparticles. 

### 3.2. Phantom

**Figure 1.**
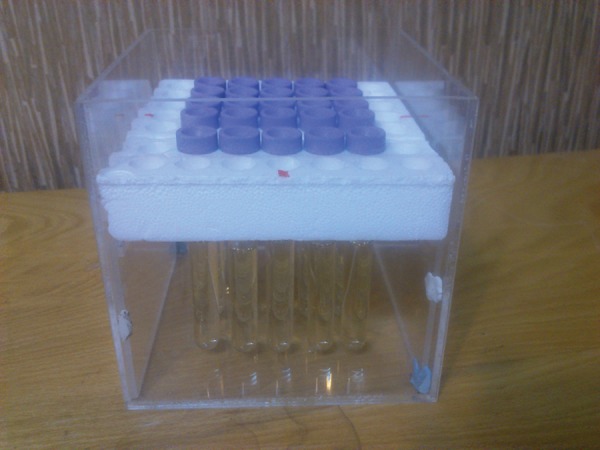
Different concentrations of iron oxide nanoparticles placed inside the phantom

A perspex phantom was designed with dimensions of 13 × 13 × 13 cm for the placement of glass vials (internal diameter = 15 mm) filled with various concentrations of iron oxide nanoparticles (Figure 1). The vials were set vertically, and the axes of the vials were perpendicular to the image plane (coronal image). The phantom consisted of 25 vials with different concentrations of nanoparticles that were carefully placed in the center of the standard clinical head coil. The vials in the phantom with a constant concentration were placed in exactly the same positions of the vials with different concentrations for the measurement of coil non-uniformity without removing the phantom from the coil.

### 3.3. Image Acquisition

MR imaging was performed using a 1.5 T MR system (MAGNETOM Vision, Siemens, Avanto, Germany) with maximum amplitude of 33 mT/m and maximum slew rate of 125 T/m/s. Non-uniformity of the coil is a major challenge in MRI that leads to image artifacts and unreliability of SI measurements (21); therefore, to measure the accurate SI of an image, the response of the RF coils should be uniform. For this purpose, the vials of constant concentration of iron oxide nanoparticles (50 µmol Fe/L) were prepared and used to measure coil non-uniformity. MRI is unable to measure the concentration of the contrast agent directly. Since there is a linear relationship between the concentrations of the contrast agent and SI, the relative changes in SI can be used instead of concentration (dose) for perfusion measurement. For this study, IR Turbo-FLASH pulse sequence was applied. In addition, protocol parameters were TR, 416 ms; TE, 1.69 ms; Tis, 100-400 ms with increments of 20 ms;matrix size, 90×128; slice thickness,10 mm; flip angle, 15º; and number of excitations (NEX) of 1.

### 3.4. Image Analysis

After MR imaging, the image data with DICOM format were transferred from the MR scanner to a personal computer. The image processing software interactive data language (IDL, Research Systems, Inc. http://www.rsinc.com) was used for data analysis. The programs were written using the IDL for obtaining correction factors of the coil non-uniformity, drawing the curves of SI versus concentration and SI versus TI, and finding the maximum linear relationship between SI and TI regarding R^2^ of 0.99. A region of interest (ROI) with 9 innermost pixels was placed in the center of each vial for each series of data and the SI of the ROIs was estimated using the IDL software. The correction factors of the coil non-uniformity were calculated and the SI of the vials with various concentrations was multiplied by these factors to obtain the corrected SI. Calculation of SI was accomplished based on Equation 1 presented by Nazarpoor (22).

Equation 1. $$S(t)=S_{0}(1-2\exp\left(-T_{i}(\frac{C(t)}{K}+\frac{1}{T_{1\mathrm{pre}}})\right)+\exp(-T_{R}(\frac{\mathrm{C(t)}}{K}+\frac{1}{T_{1\mathrm{pre}}})))$$

Where S(t) is the SI after administration of contrast agent, S0 is the observed SI in the absence of any contrast agent, C(t) is the concentration of the contrast agent at time t, T_1Pre_ is the longitudinal relaxation time before contrast application and K is a constant that depends on the contrast agent. According to this equation, T_2_ effect is negligible in high concentrations of the contrast agent. Then, the curves related to SI versus different concentrations of the nanoparticles and maximum SIs versus TIs were plotted. In addition, the concentration that gives the maximum SI with linear relationship was determined for different TIs using R^2^ of 0.99.

### 3.5. Calculation of the Injection Dose of the Contrast Agent

In order to use the results of this in vitro study for an in vivo condition, calculation of the suggested injection dose based on the in vitro results was performed using the following equation derived from Chambon et al. (15) (Equation 2).

Equation 2. $$\mathrm{ED}={\mathrm{ID.e}}^{\frac{\mathrm{-t}}{\frac{T_{1}}{2}}}$$

Where ED is the effective dose (dose remaining in the plasma at the time of imaging), ID is the injected dose, t is the time after intravenous injection of the nanoparticles and T1/2 is the half-life of the nanoparticles in plasma.

## 4. Results

**Figure 2.**
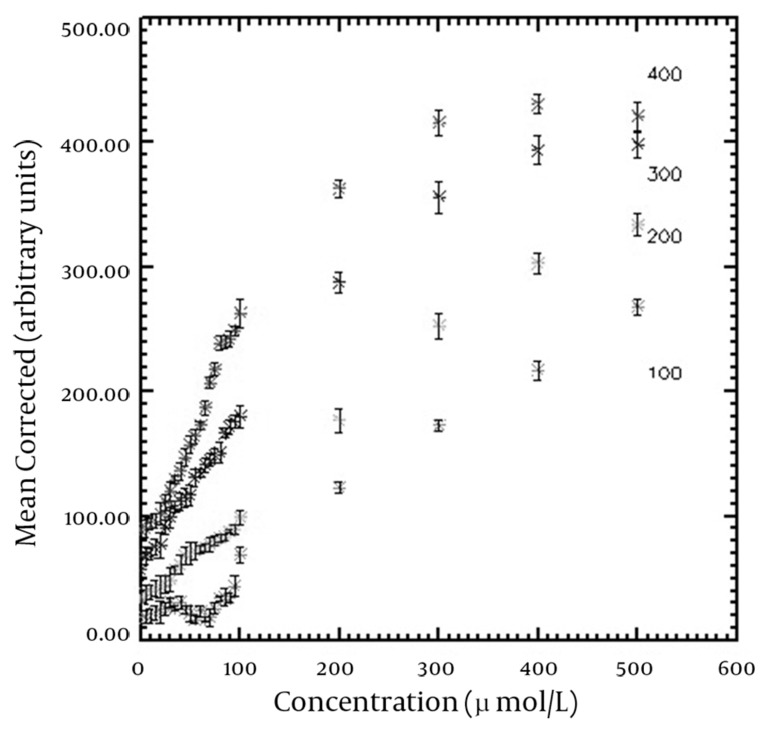
SI versus different concentrations of nanoparticle curves for typical TIs of 100, 200, 300 and 400 ms

**Figure 3.**
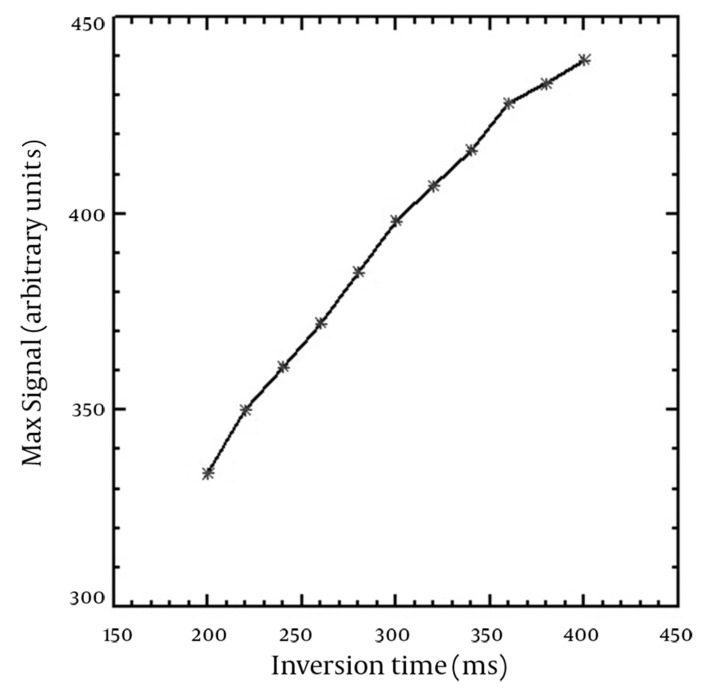
The correlation between TI and maximum SI resulted from nanoparticle concentrations

The T_1_-shortening effect was seen for all corrected SI versus concentration curves of the applied TIs. In addition, the T_2_-shortening effect appeared at high concentrations for TIs of 360-400 ms. T_1_ recovery was not seen for TIs of 100-180 ms on the horizontal axis (Mxy) and the full T_1_ recovery started from TI of 200 ms. The maximum SI resulted from the nanoparticles was obtained in the highest used concentration (500 µmol Fe/L) for TIs of 180-320 ms, while there was a slight SI difference between concentrations of 400 and 500 µmol Fe/L for TIs between 340-400 ms. The maximum SI for higher TIs was seen in 400 µmol Fe/L. SI versus nanoparticle concentration curves for typical TIs of 100, 200, 300 and 400 ms are demonstrated in Figure 2. The figure shows that the relationship between SI and concentration is linear at low concentrations. Table 1 shows the maximum linear relationship between SI and the concentrations of the nanoparticles that gives R^2^ equal to 0.99 for different TIs. The maximum linear relationship between SI and nanoparticle concentration with R^2^ of 0.99 was seen up to 239.16 µmol Fe/L for the short TI (200 ms) and 76.83 µmol Fe/L for the long TI (400 ms). Decreasing the concentration of nanoparticles that led to the maximum SI was seen by increasing the TI values. Figure 3 indicates the maximum SI versus different TIs. SI increased by increasing TI for T_1_-weighted images. Among the different utilized TIs, maximum SI was observed for TI of 400 ms. Since T_1_ recovery was not seen for the TIs of 100-180 ms on the Mxy axis, the figure started from TI of 200 ms.

The maximum SI resulted from the nanoparticles concentration and the maximum linear relationship between SI and concentrations of the nanoparticles was seen on the curves. The error bars show the standard deviation of each vial.

**Table 1.** The Maximum Linear Relationship Between SI and the Concentrations of the Nanoparticles that Gives R^2^ of 0.99 for Different TIs

Inversion Time, ms	Concentration, µmol Fe/L
**200**	239.16
**220**	210.46
**240**	186.03
**260**	164.62
**280**	152.48
**300**	139.98
**320**	128.17
**340**	118.83
**360**	109.34
**380**	93.12
**400**	76.83

The maximum SI was obtained using the highest applied TI (400 ms). The figure was plotted for TIs with T_1_ recovery.

## 5. Discussion

According to recovered curves, at the beginning of all curves (low concentrations), there is a linear relationship between SI and the concentration of the nanoparticles that becomes nonlinear at high concentrations. Due to this fact, the concentration of nanoparticles for this linearity should be determined for the calculation of SI from the concentration. The concentration of nanoparticles that leads to the highest SI was seen in a high TI of 400 ms. Therefore, it is possible to obtain maximum SI at high TIs using a lower concentration of nanoparticles. This finding is very important for in vivo studies, where the administered concentration (injected dose) of the nanoparticles as a contrast agent should be accurately determined. 

The linear relationship between the concentration of SPIO nanoparticles (AMI-25) and SI was studied by Canet et al. (16). They only used TI of 300 ms for the study. Their results showed the linear relationship between SPIO concentration and SI for concentrations ≤ 200 µmol Fe/L, while it was ≤ 139.98 µmol Fe/L in our study for the same TI. Although in both studies the same pulse sequence was applied, different sizes of nanoparticles (80-150 nm for Canet et al. study and 20 nm for our study) led to different results. According to a study conducted by Chambon et al., in similar concentrations, USPIO nanoparticles produced more SI than SPIO particles because of their higher T_1 _relaxivity. Therefore, the maximum SI that had a linear relationship with the concentration of the nanoparticles was obtained for lower concentrations compared to the study carried out by Canet et al. In our study, the maximum non linear SI was seen in the concentration of 400 µmol Fe/L for TIs of 360-400 ms and it was 500 µmol Fe/L for lower TIs. Because SI does not change considerablyin TI concentrations between 260 and 340 ms, the SI difference between nanoparticle concentrations of 400 and 500 µmol Fe/L is very small and it may be negligible. Chambon et al. considered signal enhancement of different concentrations of USPIO nanoparticles for an in vitro study using spin echo pulse sequence with different TRs and TEs (15). They reported a considerable signal enhancement at low concentrations of USPIO nanoparticles using TR of 500 ms and TE of 22 ms. Their results showed a maximum USPIO nanoparticle enhancement at 200 µmol Fe/L applying these parameters using a 1.5 T MRI system. They also found maximum enhancement with the concentration of 400 µmol Fe/L using TR of 160 ms and TE of 20 ms that is in agreement with our study. These findings demonstrate that applying different sequences can lead to different results; however, it can be noticed that the sequence parameters have an influencing impact on the achieved results. There was no explanation about the linear relationship between the nanoparticle concentration and SI in their study. Moreover, it should be mentioned that the mean nanoparticle size was 20 nm for both studies.

As seen in Table 1, the concentration of nanoparticles that led to the maximum SI in linear pattern decreased by increasing TI values and the minimum concentration (76.83 µmol Fe/L) was obtained using TI of 400 ms. Therefore, placing the concentration of 76.83 µmol Fe/L as ED in equation 2, and 6 hours as the plasma half-life of USPIO nanoparticles with carboxydextran coating and a size of approximately 20 nm (23), the concentration of 76.98 µmol Fe/L (~77 µmol Fe/L) was obtained as the injected dose (ID). This value for injection dose is equal to 4.22 mg Fe/kg in clinical studies. Other researchers reported an appearance of susceptibility artifact with a dose of 4 mg Fe/kg of NC 100150 nanoparticles (24). Chambon and colleagues found a difference between maximum enhancements observed for in vitro and in vivo studies (15). The researchers explained an in vivo magnetic susceptibility-related phenomenon caused by flow mechanisms. Magnetic susceptibility is produced by the movement of magnetic particles in the field in different directions from the phase encoding (15). Considering the susceptibility artifact caused by the effect of iron oxide nanoparticleson image quality, it seems that in vivo application of the dose of 4.22 mg Fe/kg has clinical limitation. However, the differences between the two results are negligible and the probability of susceptibility artifact can be reduced using small TEs.

Reimer et al. used 20 nm nanoparticles with carboxydextran coating for myocardial perfusion and MR angiography of the chest (17). They injected four doses of nanoparticles including 5, 10, 20, and 40 µmol Fe/kg intravenously and performed MR imaging during the first pass and the equilibrium phase and reported capability of the highest nanoparticles dose to depiction of blood vessels in the chest at the first pass angiography and for cardiac perfusion. The pulse sequence and imaging parameters used in our study were similar to their study. Nonetheless, they used a TI of 200 ms for cardiac perfusion imaging, but we performed the study using different TIs to evaluate the TI effect on the relationship between different concentrations of nanoparticles and SI values. Our results show that obtaining higher SI values using larger concentrations than 40 µmol Fe/kg for TI of 200 ms is possible. However, they did not use higher doses than 40 µmol Fe/kg.

In this study, IR Turbo-FLASH sequence was used for MR study. Turbo-FLASH sequence is one of the ultra-fast pulse sequences of MRI that is used for MR angiography (16, 17). In this sequence, the T_1_ effect of nanoparticles appears on the images because of applying short TR and TE. Therefore, an improvement of SI is achieved in this pulse sequence (25).

The selection of imaging parameters for our study was based on a study conducted by Reimer et al. on the myocardial perfusion and MR angiography of the chest. In our study, the short echo time of 1.69 ms was selected to obtain further increase in SI and to minimize the susceptibility artifact. Using short TE can also lead to good linear correlation between the nanoparticle concentration and the SI (26).

The size of the nanoparticles and their coating material have important roles in biological properties of the contrast agent. A study showed that allergic reactions were decreased using carboxydextran coating of the nanoparticles (27). In this study, we used carboxydextran-coated iron oxide nanoparticles with a hydrodynamic size of 20 nm as a MR contrast agent. These nanoparticles have a prolonged blood half-life because of their very small size decreasing their uptake by liver. This property provides adequate time for serial MR imaging of the vessels in MR angiography. Other studies demonstrated an excellent T_1_ effect using nanoparticles sized approximately 21 nm (28). We should mention that our study was an in vitro study so the results need to be evaluated further in other in vivo studies for practical applications. 

In the current study, the maximum SI and linear relationship between different concentrations of USPIO nanoparticles with SI was evaluated applying different TIs. TI is an important parameter to consider in the relationship between SI and the nanoparticle concentrations. The minimum concentration that leads to maximum SI obtained using the highest applied TI. An increase in TI leads to a decrease in the range of linearity. Considering the maximum linear relationship between SI and the concentration of nanoparticles can help to determine the minimum concentration that leads to the maximum SI of the contrast agent and therefore, it will improve the signal-to-noise ratio of T_1_-weighted MR images. This study was performed using a routine 1.5 T MRI system and the selected applied parameters were based on previous studies of other researchers active in the field of clinical studies. Therefore, the results can be applied for MR cardiac perfusion in patients for future works.
